# Biological Reinforced Concrete for Cartilage Repair With 3D Printing

**DOI:** 10.1002/advs.202416734

**Published:** 2025-02-25

**Authors:** Yuewei Chen, Tao Fu, Zhongfei Zou, Yanming Liu, Jianguo Zhu, Binhong Teng, Ke Yao, Haibin Li, Jiachun Li, Zhijian Xie, Yong He

**Affiliations:** ^1^ School of Mechanical Engineering Guizhou University Guiyang 550025 China; ^2^ State Key Laboratory of Fluid Power and Mechatronic Systems & Liangzhu Laboratory School of Mechanical Engineering Zhejiang University Hangzhou 310027 China; ^3^ Department of Oral and Maxillofacial Surgery The Second Affiliated Hospital of Zhejiang University School of Medicine, School of Stomatology and Key Laboratory of Oral Biomedical Research of Zhejiang Province Hangzhou Zhejiang 310000 China; ^4^ Stomatology Hospital, School of Stomatology, Zhejiang University School of Medicine, Zhejiang Provincial Clinical Research Center for Oral Diseases, Key Laboratory of Oral Biomedical Research of Zhejiang Province Cancer Center of Zhejiang University, Engineering Research Center of Oral Biomaterials and Devices of Zhejiang Province Hangzhou 310000 China; ^5^ Department of Orthodontics The Second Affiliated Hospital of Zhejiang University School of Medicine Hangzhou Zhejiang 310000 China; ^6^ Department of Urology Guizhou Provincial People's Hospital The Affiliated Hospital of Guizhou University Guiyang Guizhou 550002 China; ^7^ School of Mechanical Engineering Guizhou Institute of Technology Guiyang 550003 China

**Keywords:** 3D‐printed ultrafine fiber networks, biohydrogels, biological reinforced concrete, cartilage, extracellular matrix

## Abstract

The development of biomimetic cartilage constructs (BCCs) with natural extracellular matrix (ECM) microenvironments and topological cues to accelerate the reconstruction of natural articular cartilage (NAC) after injury is challenging due to its complex structure, low cellular content, and less vascularity. Inspired by concrete rebar structure, a biomimetic cartilage named “biological reinforced concrete” is fabricated, with collagen fiber orientation transitioning from parallel to perpendicular, replicating the ECM microenvironments and complex construct of NAC. 3D‐printed ultrafine fiber networks (UFNs) served as a degradable “biorebars”, while a hybrid biohydrogel acted as “biocement”. The stem cells are utilized as “bioactive aggregates”. The biocement is developed by combining and screening various biohydrogels to mimic an ECM microenvironment conducive to the formation of NAC. By adjusting the fiber scale and spacing of the UFNs, the mechanical properties of the biomimetic cartilages are controlled to resemble those of NAC. Additionally, the UFNs guided the directed growth of cells and the orderly secretion of ECM. Subsequently, the developed BCCs are implanted into an osteochondral defect, and after 4 months, they successfully reconstructed the complex structure of cartilage with mechanical properties closely resembling those of NAC. The biological reinforced concrete offers a customizable and universal strategy for tissue regeneration.

## Graphical abstract

Inspired by concrete rebar structures in engineering, the concept of biological reinforced concrete uses 3D‐printed ultrafine fiber networks (UFNs) as biodegradable “biorebars”， hybrid biohydrogels as “biocement”， and stem cells as “bioactive aggregates” to fabricate biomimetic cartilage. Screening various biohydrogels mimics the cartilage extracellular matrix (ECM) microenvironment. Tuning the UFNs orientation and spacing matches the ECM topology and cartilage mechanical properties.

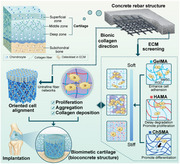

## 1 Introduction

Articular cartilage injury is common, resulting in the loss of partial joint function and significantly impacting quality of life. Notably, severe cartilage injury can cause excruciating pain and penetrate deep into the subchondral bone, and left untreated, can develop into osteoarthritis or disability.^[^ ^1^, ^2^ ^]^ However, repairing or reconstructing articular cartilage poses significant challenges due to low cell count, less vascularity, absence of lymphatic vessels, lack of neural innervation, and poor self‐repair ability. Although many strategies have been developed for cartilage repair,^[^ ^3^, ^4^ ^]^ such as microfracture and matrix‐assisted chondrocyte implantation, these methods can only regenerate fibrous cartilage with poor mechanical properties, resulting in unsatisfactory repair effects.^[^ ^5^ ^]^ Therefore, developing a biomimetic natural extracellular matrix (ECM) cartilage construct to accelerate the reconstruction of functional articular cartilage is a promising strategy in clinical practice.^[^ ^6^, ^7^, ^8^, ^9^, ^10^ ^]^

The physiological structure of articular cartilage is highly anisotropic,^[^ ^11^ ^]^ exhibiting regional variations as it extends from the articular surface to the subchondral bone, forming a zonal structure with three specific regions: superficial, middle transitional, and deep zones.^[^ ^12^ ^]^ Each region exhibits unique ECM characteristics, with the most significant difference being the alignment of collagen fiber networks, which influences the mechanical properties of each area.^[^ ^13^ ^]^ Notably, the distribution and alignment of cells differ in each region.^[^ ^14^ ^]^ In the superficial zone, collagen fibers are arranged in rows oriented parallel to the articular surface.^[^ ^15^ ^]^ The cells appeared to be more numerous and are arranged in a horizontally aggregated formation.^[^ ^16^ ^]^ In the middle transitional zone, the collagen fibers are larger in diameter and inclined toward the articular surface, providing a mechanical cushion between the cartilage and subchondral bone.^[^ ^17^, ^18^ ^]^ In the deep zone, the collagen fibers are oriented perpendicular to the articular surface, and the elastic modulus is several orders of magnitude higher than those of the other two regions.^[^ ^19^ ^]^ Notably, the ECM of articular cartilage is dominated by collagen and mainly includes organic aggregates like hyaluronic acid (HA) and negatively charged glycosaminoglycans, such as chondroitin sulfate.^[^ ^17^, ^20^ ^]^ Therefore, the design and fabrication of biomimetic cartilage with ECM constructs pose considerable challenges. In recent years, tissue engineering strategies have developed various constructs, including single‐phase and multiphase scaffolds, to mimic the complex structure and biomechanics of cartilage ECM.^[^ ^21^, ^22^, ^23^, ^24^, ^25^, ^26^, ^27^, ^28^ ^]^

Biohydrogels, such as gelatin methacryloyl (GelMA) and hyaluronic acid methacryloyl (HAMA), have emerged as a promising encapsulation material for cells, as well as mimickers of the ECM microenvironment, for 3D culturing of cells and regulating cell behavior, such as migration, proliferation, and differentiation.^[^ ^29^, ^30^, ^31^, ^32^ ^]^ Notably, The stiffness of the biohydrogel also plays a key role in cell differentiation.^[^ ^33^, ^34^ ^]^ For example, soft matrices (stiffness <10 kPa) are widely used to induce stem cell differentiation for neurogenesis or vascularization, whereas stiffer matrices (>25 kPa) are used for osteogenesis.^[^ ^35^, ^36^ ^]^ The biohydrogels such as GelMA, HAMA, and chondroitin sulfate methacryloyl (ChSMA), are derived from secondary products of the ECM components.^[^ ^20^ ^]^ However, biohydrogels that achieve high cell viability and promote ECM deposition often exhibit low mechanical properties, and replicating the mechanical characteristics of natural cartilage is challenging. Furthermore, at the tissue level, biomaterials as implanted scaffolds must match the mechanical properties of the host tissue.^[^ ^37^ ^]^ Notably, the alignment of collagen fibers undergoes a transition from parallel to the arthrosis surface in the superficial zone to an inclined orientation in the middle transitional zone, where the collagen fibers are relatively thicker. This intricate structure presents significant challenges for the reconstruction of natural cartilage using only the biochemical microenvironment of hybrid biohydrogels due to some limitations: 1) poor mechanical performance; 2) difficulty in replicating the anisotropy of natural cartilage; and 3) absence of essential topological cues to guide cell migration and the directed deposition of ECM.

However, developing biomimetic cartilage constructs that possess the biochemical microenvironment and topological cues of the ECM is challenging, especially when aiming for optimal mechanical properties. Therefore, perfect biomimetic cartilage constructs (BCCs) must have the following features: 1) mechanical properties matching natural articular cartilage; 2) the topological structure and scale of the fiber network can mimic the alignment of collagen fibers in natural articular cartilage, guiding directional cellular migration and ECM deposition; 3) a biochemical microenvironment mimicking cartilage ECM that promotes accelerated secretion of ECM in vivo; and 4) degradability. 5) Integration of the regenerated tissue with the surrounding tissue. Numerous researchers employed solution electrospinning technology to fabricate nanofiber membranes that mimicked ECM structure. However, the drawbacks of nanofiber membranes, including high porosity and small pore size, result in inadequate cell infiltration. Additionally, the inability to deposit fibers in a controlled manner to create topological structures that simulate ECM structure is the most significant limitation of solution electrospinning technology. Melt electrowriting (MEW) is a high‐precision additive manufacturing technology that enables controlled fiber deposition, and also controls fiber diameter (800 nm‐150 µm).^[^ ^38^ ^]^ Because this technology can print ultrafine fiber networks (UFNs) with various topological structures, it can replace the alignment and function of ECM collagen fibers in a controllable manner to regulate cell behavior, resulting in widespread use in tissue engineering.^[^ ^39^, ^40^, ^41^, ^42^ ^]^

**Figure 1:**
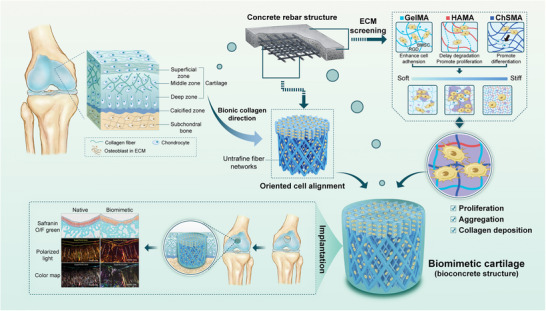
Schematic illustration of biological reinforced concrete for cartilage repair.

To address these challenges, we proposed the concept of biological reinforced concrete, inspired by the idea of concrete rebar structure. The BCCs were designed and fabricated to accelerate the reconstruction of natural cartilage (**Figure** **1**), and consist of three components: “biocement”, “degradable biorebar”, and “activated stone”. The biocement was screened and formulated three types of biohydrogels that mimic the 3D microenvironment of cartilage ECM to accelerate ECM deposition. The 3D‐printed UFNs served as biorebars. The activated stone was made up of stem cells that were used to promote tissue regeneration. The BCCs were constructed by integrating two layers of biorebar structures with biocement. Based on the alignment of collagen fibers in various regions of natural cartilage, the superficial zone of the biomimetic cartilage comprised orthogonal UFNs. The middle transitional zone was constructed as a cylinder using multiscale rhombus UFNs rolled into, with fine fibers guiding cellular orientation and coarse fibers providing mechanical support. The mechanical properties of BCCs were fine‐tuned to align with the modulus of natural cartilage by modifying the fiber spacing and diameter of the UFNs. The biological functions of biorebar guided the directional migration of cells and induced orderly secretion of ECM. The BCCs were implanted into an osteochondral defect, which successfully reconstructed the complex structure of natural cartilage in vivo. The results indicated that biological reinforced concrete has promising clinical application prospects in tissue engineering.

## 2 Results and Discussion

### 2.1 Design of “Biocement” to Simulate the Cartilage ECM 3D Microenvironment

Cells secrete and synthesize the ECM, which regulates cell behavior, including proliferation and differentiation. The interaction forms a dynamic system between cells and ECM, which is crucial for tissue formation, repair, and functional maintenance. The ECM not only provides structural support but also participates in the regulation of cellular behaviors, such as proliferation, differentiation, migration, and apoptosis. Therefore, many new functional biomaterials and modified classical hydrogel materials have been used to simulate the 3D biochemical microenvironment of cartilage ECM, to promote cartilage regeneration.^[^ ^43^, ^44^, ^45^ ^]^ The intricate biochemical microenvironment of cartilage ECM poses significant challenges for mimicking in vitro. However, biologically derived hydrogels, such as collagen and glycosaminoglycans, which can mimic the chemical composition of natural ECM, show excellent biocompatibility and maintain the biochemical properties of ECM, making them widely used in tissue engineering and regeneration. The cartilage ECM primarily comprises collagen, with negatively charged sulfated glycosaminoglycans attached to the HA skeleton, forming aggregated protein polysaccharides. Drawing inspiration from the composition of cartilage ECM, a gelatin‐based hybrid biohydrogel system was designed as biocement. This biocement uses GelMA as the skeleton and regulates the ratio of HAMA and ChSMA to create a 3D microenvironment that is mechanically and biochemically adjustable, effectively mimicking the ECM 3D microenvironment.

The GelMA biohydrogel is a derivative of collagen, inheriting many superior biological properties of collagen, including the promotion of cell adhesion and growth through the Arginine‐Glycine‐Aspartic Acid (RGD) peptide and enzymatic degradation ability.^[^ ^46^ ^]^ In a hybrid biohydrogel system, GelMA provides bioactive adhesion sites RGD to enhance biocompatibility, mediating cell‐matrix interactions. However, GelMA has poor mechanical properties and is easily degraded by cells,^[^ ^47^ ^]^ making it difficult for collagen alone to promote cartilage matrix formation. HAMA has excellent biological functions, such as regulating cell proliferation, and differentiation and promoting cartilage formation.^[^ ^48^ ^]^ The introduction of HAMA can alleviate the degradation rate of the hybrid system and increase mechanical stability.^[^ ^49^ ^]^ Additionally, GelMA complements the poor cell adhesion properties of HAMA. ChSMA provides robust biochemical cues to guide and promote cartilage formation by mesenchymal stem cells.^[^ ^50^ ^]^

In the human body, almost all somatic cells are in a 3D matrix containing a complex array of ECM components. The ECM, predominantly composed of 3D networks and enriched with bioactive signaling molecules, supports cell survival and determines cell fate.^[^ ^51^ ^]^ Biomaterial design should consider the synergistic interactions among various components, mimicking ECM to preserve physiological functions and facilitate their precise functional execution. The communication between cells and the ECM 3D microenvironment is regulated through biochemical and mechanical signals. These signals are crucial in determining cell fate, including directing differentiation and development.^[^ ^52^, ^53^, ^54^ ^]^ Therefore, screening a 3D microenvironment that can effectively induce cellular differentiation is key to maximizing the efficacy of cells.

**Figure 2:**
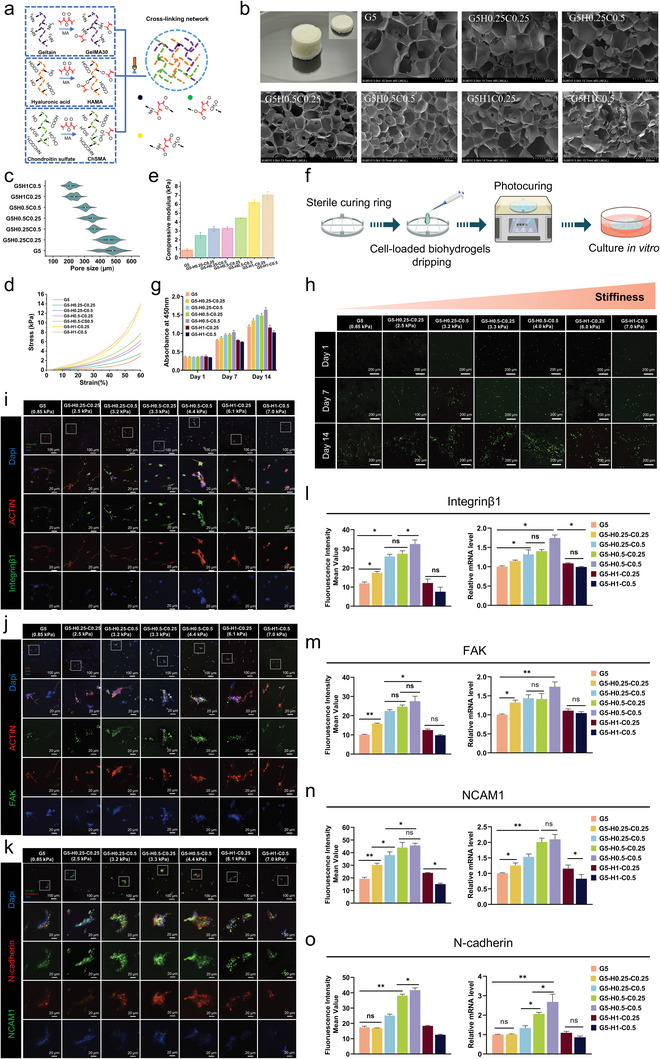
Proliferation, migration, and aggregation potential of 3D‐cultured hBMSCs in vitro. a) Diagram of the internal network of a hybrid biohydrogel. b) SEM analysis of the internal structure of freeze‐dried hybrid biohydrogel. c) Pore sizes, d) Compressive stress‐strain curves e) and Compressive modulus of hybrid‐biohydrogels group. f) Workflow of 3D‐cultured hBMSCs on the sterile curing ring. g) CCK‐8 analysis of 3D‐cultured hBMSCs proliferation in hybrid‐biohydrogel groups for 1, 7, and 14 days. h) Live/dead staining of 3D‐cultured hBMSCs in hybrid‐biohydrogel groups for 1, 7, and 14 days (green: live cells; red: dead cells). i) Confocal images of integrin‐β1 expression (red: actin, green: integrin‐β1, and blue: nucleus), j) FAK expression (red: actin, green: FAK, and blue: nucleus), k) NCAM1 and N‐cadherin expressions in 3D‐cultured hBMSCs (blue: nucleus, green: NCAM1, and red: N‐cadherin) in hybrid‐biohydrogel groups after 7 days. l–o) Mean fluorescence intensity and mRNA expression of integrin‐β1, FAK, NCAM1, and N‐cadherin after 3D culture. Data are means ± SD. *p < 0.05 and **p < 0.01.

In the natural 3D environment, cells respond to mechanical stress that is transmitted by the matrix. Therefore, the biological behavior of cells is intricately linked to the stiffness and biological properties of the matrix. Although many studies have investigated cell responses to matrix stiffness in 2D culture models, where altering matrix stiffness regulates cell fate, it is important to note that cells in organisms exist within a complex and dynamic 3D ECM. This complexity underscores the high demand for tunable mechanical properties of hybrid biohydrogel matrix. In the hybrid biohydrogel system, our approach was to fix the concentration of GelMA as a skeleton and adjust the concentrations of HAMA and ChSMA to regulate overall stiffness, thereby controlling cell fate. To create a 3D microenvironment with adjustable stiffness and biochemical cues for simulating the ECM and promoting cartilage formation, three types of biohydrogels were developed to produce hybrid‐biohydrogel 3D networks: GelMA, HAMA, and ChSMA. The methacryloyl (MA) substitution rate of GelMA was 30% to expose more RGD adhesive sites in the hybrid‐biohydrogel network. The molecular chains of the three biohydrogels were intricately intertwined, creating a stable mechanical and biochemical 3D microenvironment through MA covalent bonds (**Figure** **2**a). To investigate the synergistic promotion of cartilage formation through stiffness and biochemical cues in a 3D microenvironment and accelerate cartilage matrix deposition, a gradient of matrix stiffness was designed by adjusting the concentrations of HAMA and ChSMA. Therefore, seven formulations were developed to offer a wide range of adjustable moduli (**Table** **1**).

**Table 1:** Experimental formulations.

Group Element [%, w/v]	1	2	3	4	5	6	7
GelMA HAMA ChSMA	5	5	5	5	5	5	5
	0	0.25	0.25	0.5	0.5	1	1
	0	0.25	0.5	0.25	0.5	0.25	0.5

The duration of light curing significantly impacts biohydrogel stiffness. In low‐concentration biohydrogels, the insufficient curing time can impede the liquid‐to‐solid transition. Therefore, it is necessary to test the gelation time of hybrid biohydrogels. The light rheology of seven hybrid biohydrogels under a light intensity of 30 mW cm^−2^ revealed that the light exposure time for the gelation point decreased with increasing the combined concentration of HAMA and ChSMA, whereas the storage modulus displayed an opposite trend (Figure s1a,b, Supporting Information). Given that the light exposure time of the gelation point was longest for G5 at 27.9 s, and ≈7.5 s for the highest concentration (G5‐H1‐C0.5) (Figure s1c, Supporting Information). Therefore, all hybrid‐biohydrogel groups were subjected to 30 s of light curing to ensure complete gelation.

As shown in Figure 2b, analysis of the seven freeze‐dried hybrid biohydrogels using scanning electron microscopy (SEM) revealed an uneven distribution of pore sizes. Notably, an increase in the combined concentrations of HAMA and ChSMA led to a noticeable reduction in pore size. The G5 exhibited the largest average pore sizes (≈500 µm), whereas the remaining hybrid biohydrogels displayed relatively similar pore sizes (range: 200–400 µm) (Figure 2c). To assess the matrix stiffness of the seven hybrid biohydrogels, their compressive modulus was evaluated, and a nonlinear stress‐strain curve with a distinctive J‐shaped response was observed (Figure 2d). As shown in Figure 2e, quantitative calculations of compressive modulus for the seven hybrid hydrogel groups revealed that G5 was relatively soft (modulus: 0.85 kPa), closely resembling that of brain tissue (≤1 kPa). G5 served as a framework, enabling a broad range of modulus adjustments for the hybrid biohydrogels. The compressive modulus of the hybrid biohydrogels progressively increased as the combined concentrations of HAMA and ChSMA rose, with the G5‐H1‐C0.5 exhibiting the highest value of 7 kPa. These findings demonstrated the hybrid‐biohydrogel system's ability to control stiffness within a range of 0.8–7.0 kPa.

During chondrogenic differentiation and regeneration, a series of cellular behaviors are exhibited by mesenchymal stem cells, such as proliferation, migration, aggregation, and differentiation. Additionally, the subsequent synthesis and deposition of the cartilage matrix are critical for cartilage formation.^[^ ^51^, ^55^ ^]^ These steps are not isolated events but interact and depend on each other through a complex and precisely regulated 3D network. Notably, after cell proliferation and migration, the aggregation of human bone marrow‐derived mesenchymal stem cells (hBMSCs）enhances intercellular contact and interaction, which is crucial for forming a stable cell colony. Such a colony not only helps maintain an appropriate cellular microenvironment but also plays significant role in driving hBMSC differentiation along the chondrogenic pathway and in the deposition of the cartilage matrix.^[^ ^56^, ^57^ ^]^ In designing biohydrogels that promote hBMSC differentiation and cartilage regeneration, these key cellular behaviors must be thoroughly considered to ensure that the simulated 3D microenvironment supports cartilage regeneration.

First, high cell viability must be ensured under 3D‐cultured conditions. The study initially evaluated 3D‐cultured hBMSCs in hybrid biohydrogels using live/dead staining and a proliferative capacity test. The hBMSC‐laden biohydrogels were uniformly dispensed onto a sterilized curing ring and photocured. The curing ring served as a support to maintain the structural stability of the biohydrogel (Figure 2f). Cytotoxic effects were not observed across all groups (Figure 2h). Cell viability was well‐maintained, with only a minimal number of dead cells detected by day 14. As shown in Figure 2g, analysis of cell proliferation with the CCK‐8 assay revealed no significant differences in optic density values among the groups on day 1. When the culture period was extended, all groups showed an increase in proliferation. Notably, after 14 days of continuous culture, the number of cells in the G5‐H0.5‐C0.5 biohydrogel was increased, indicating the effectiveness of this biohydrogel in supporting cell growth. Unexpectedly, a noticeable decline was observed in the G5‐H1‐C0.25 and G5‐H1‐C0.5 groups. In 3D gel matrices, cells experience moderate matrix forces in matrices with optimal crosslinking strength, which facilitates cell proliferation, adhesion, and migration. However, excessive matrix forces can inhibit cell behavior, and high stiffness may restrict the dynamic microenvironment remodeling capabilities of the cells.^[^ ^52^, ^58^ ^]^

Cells encapsulated in 3D matrices interact with ECM ligands, such as integrins, which are transmembrane receptors that mediate cell‐microenvironment communication. Enhanced integrin expression in biohydrogel matrices indicates the initiation of cell proliferation and differentiation trajectory.^[^ ^51^, ^59^, ^60^ ^]^ Hence, after 7 days of 3D culture, the expression of integrin‐β1 was investigated across seven hybrid‐biohydrogel groups. As shown in Figure 2i,l, compared with the G5 group, the expression of integrin‐β1 was upregulated in the G5‐H0.25‐C0.25, G5‐H0.25‐C0.5, G5‐H0.5‐C0.25, and G5‐H0.5‐C0.5 groups. Notably, the G5‐H0.5‐C0.5 group exhibited the most significant increase in integrin‐β1 expression, suggesting the highest activity of integrin‐β1 under this biohydrogel condition. Conversely, the G5‐H1‐C0.5 group showed relatively weaker integrin‐β1 expression, which can be attributed to specific cell‐matrix interactions in this microenvironment.

Subsequently, the abilities of the hybrid biohydrogel matrices to regulate the migration and aggregation of hBMSCs were studied by evaluating the expression of the cell migration marker focal adhesion kinase (FAK) and aggregation markers neural cell adhesion molecule 1 (NCAM1) and neural cadherin (N‐cadherin). The upregulation of these markers is a prerequisite for cartilage formation. After 7 days of 3D culture, FAK immunofluorescence revealed that FAK manifests as bright spots in areas where the cytoskeleton adheres to the substrate (Figure 2j). The expression levels were highest in the G5‐H0.5‐C0.5 and G5‐H0.5‐C0.25 groups, whereas the lowest expression was observed in the G5‐H1‐C0.5 and G5 groups (Figure 2m). As shown in Figure 2k, double immunofluorescence staining of NCAM1 and N‐cadherin showed that they did not completely overlap and their expression was most prominent in the G5‐H0.5‐C0.5 group. In addition, the fluorescence intensity of the G5‐H0.5‐C0.25 group was notable, but slightly lower in the G5‐H0.5‐C0.5 group. The expression levels in the remaining groups were lower than those in the G5‐H0.5‐C0.5 and G5‐H0.5‐C0.25 groups (Figure 2n,o). Additionally, a reduction in N‐cadherin expression was more noticeable than that of NCAM1 expression in these groups, implying that N‐cadherin was more sensitive and readily influenced by the biohydrogel matrix. As shown in Figure 2l–o, the mean fluorescence intensity of integrin‐β1, FAK, NCAM1, and N‐cadherin followed the same trend. As anticipated, by day 7, the G5‐H0.5‐C0.5 group showed significantly higher mRNA expression levels of integrin‐β1, FAK, NCAM1, and N‐cadherin compared to other groups, with increases of 1.74‐, 1.61‐, 2.09‐, and 2.68‐fold, respectively, over G5. The overall trend in each group was generally consistent with their respective immunofluorescence.

These findings further confirm that among the seven screened hybrid‐biohydrogel groups, the G5‐H0.5‐C0.5 group, due to the synergistic effects of its components, provides relatively optimal biochemical cues and mechanical properties, creating a more suitable and definitive matrix 3D microenvironment for stem cells. This microenvironment is particularly advantageous in promoting vital cellular events, including hBMSC proliferation, migration, and aggregation.

### 2.2 Chondrogenesis and Matrix Secretion of 3D‐Cultured hBMSCs In Vitro

**Figure 3:**
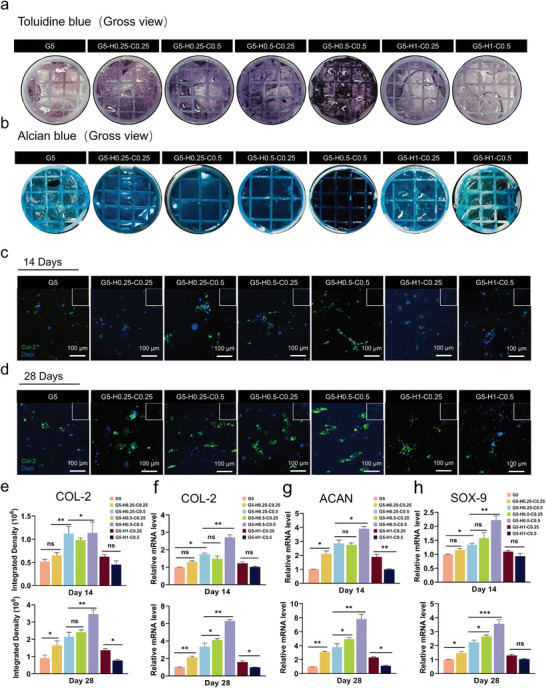
Chondrogenesis and matrix secretion of 3D‐cultured hBMSCs in vitro. a) Toluidine blue staining and b) Alcian blue staining after 3D‐cultured hBMSCs with chondrogenic medium for 28 days. c, d) Gross view and magnified images of COL‐2 expression in each group after 14 and 28 days of chondrogenic induction (green: COL‐2, blue: nuclei). e) Quantitative analysis of the COL‐2 immunofluorescence integrated density of each group at 14 and 28 days. f–h) Expression of chondrogenic genes (COL‐2, ACAN, SOX‐9) in 3D‐cultured hBMSCs within hybrid‐biohydrogel matrices at 14 and 28 days. Data are shown as mean ± SD. *p < 0.05, **p < 0.01, and ***p < 0.001.

In the optimal scenario, stem cells undergo proliferation, migration, and aggregation, and subsequently, guided by specific cues, differentiate toward chondrogenesis. This process involves the secretion of a cartilage matrix, culminating in tissue remodeling.^[^ ^61^ ^]^ In this section, the performance of the seven hybrid‐biohydrogel groups was examined and screened on the chondrogenesis and collagen matrix secretion of hBMSCs in vitro. First, the curing ring carrying the hBMSC/biohydrogel was transferred into a 24‐well plate and cultured using a chondrogenic induction medium. On day 28, the samples were stained with toluidine blue and alcian blue separately. In all groups, the chondrogenic differentiation of hBMSCs in the 3D microenvironment was observed (**Figure** **3**a,b), which was evidenced by positive staining with toluidine blue and alcian blue, indicating the deposition of proteoglycan‐rich, cartilage‐like ECM, and the G5‐H0.5‐C0.5 group enjoyed the darkest stains. The relative chondrogenic potential of hBMSCs within the seven groups was quantified. After 14 and 28 days of chondrogenic induction was complete, type II collagen (COL‐2) immunofluorescent staining was performed and cells were observed using confocal laser scanning microscopy (CLSM). Both cell density and the COL‐2 protein matrix secreted by cells in each of the seven groups increased over the culture period. By day 14, COL‐2 expression predominantly manifested in a scattered, punctate pattern surrounding the cells (Figure 3c). The highest COL‐2 expression (integrated density) was found in the G5‐H0.5‐C0.5 group, followed by G5‐H0.25‐C0.5, G5‐H0.5‐C0.25, G5‐H0.25‐C0.25, G5‐H1‐C0.25, G5, and G5‐H1‐C0.5. As shown in Figure 3d,e, compared with that on day 14, all groups exhibited an increase in expression area and overall fluorescence intensity of COL‐2 expression at the 28‐day mark. Notably, the G5‐H0.5‐C0.25 group surpassed the G5‐H0.25‐C0.5 group, becoming the second‐highest in expression, whereas the trends in the other groups remained unchanged.

The expression of typical chondrogenic‐related genes, COL‐2, aggrecan (ACAN), and transcription factor Sry‐related HMG box 9 (SOX‐9), was evaluated using qRT‐PCR. Overall, as shown in Figure 3f–h, the trends indicated by PCR analysis aligned with the findings from the immunofluorescence tests. The G5‐H0.5‐C0.5 group showed a relatively higher expression of chondrogenic genes than the other groups. Notably, on day 14, the relative mRNA levels of COL‐2, ACAN, and SOX‐9 in the hBMSCs of G5‐H0.5‐C0.5 were 2.72‐, 3.91‐, and 2.22‐fold higher than those in the G5 group, respectively. On day 28, the expression of COL‐2, ACAN, and SOX‐9 mRNA in the G5‐H0.5‐C0.5 group was 6.31‐, 7.80‐, and 3.56‐fold higher than in the G5 group.

To summarize, among all experimental groups, the G5‐H0.5‐C0.5 group had the most conducive 3D microenvironment for promoting chondrogenic differentiation. This outcome aligns with the results from the previous part of the study and can be regarded as a continuation and feedback of the upregulation of ECM components, cellular signal transduction receptors, and biomarkers related to cell migration and aggregation. Therefore, the G5‐H0.5‐C0.5 biohydrogel was chosen for subsequent experiments. However, the growth and migration of cells in the 3D microenvironment, which lacks topological cues, as well as the distribution and alignment of the resultant cartilage matrix, were disorganized and random. Furthermore, it is undeniable that the biohydrogels selected in this study are quite soft. The compressive modulus of the G5‐H0.5‐C0.5 biohydrogel was 4.45 kPa, which was higher than that of G5 but significantly lower than that of natural cartilage. Although biohydrogel matrices can regulate the 3D activity of hBMSCs and the deposition of collagen matrix can increase mechanical stress, a significant disparity remains compared with the higher elastic modulus required for cartilage defect implants or natural cartilage tissue.^[^ ^62^ ^]^

### 2.3 Fabrication and Mechanical Properties of Biomimetic Cartilage Constructs

**Figure 4:**
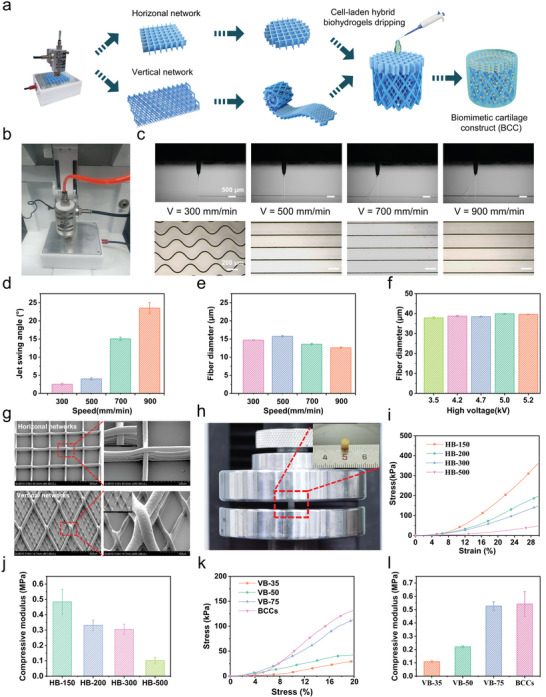
Fabrication and mechanical properties of the BCCs. a) Assembly process of BCCs. b) The MEW machine. c) Images of the swing angle of the stable jet and the deposition state of the jet at different collection speeds. d) Swing angle of the stable jet with different collection speeds. e) Diameter of deposited fibers at different speeds. f) Diameter of deposited fibers at different high voltages. g) SEM images of the orthogonal and rhombus UFNs. h) Images of the compressive modulus test. i) Compressive stress‐strain diagram. j) Compressive modulus of horizontal biological reinforced concretes. k) Compressive stress‐strain diagram. l) Compressive modulus of vertical biological reinforced concretes and BCCs.

Drawing inspiration from the morphology and alignment of collagen fibers in natural cartilage, the UFNs were integrated as topological cues. Orthogonal UFNs (horizontal networks) were utilized to simulate the parallel collagen fibers of the superficial zone of cartilage, whereas multiscale 45° rhombus UFNs (vertical networks) were coiled into a cylindrical shape to simulate the middle transitional zone (**Figure** **4**a). Ultimately, these two components were combined to create a biomimetic anisotropic collagen alignment that served as a topological cue. Subsequently, the G5‐H0.5‐C0.5 biohydrogel containing hBMSCs was poured into the topological cue to fabricate a BCC (height: 4 mm, diameter: 4 mm).

The UFNs were fabricated by custom MEW equipment using PCL material (Figure 4b). As the number of printing layers increases, the electric field strength decreases, leading to a larger swing angle of the jet and affecting the printing quality of the UFNs. To counteract this, it is necessary to increase the voltage to reduce the jet's swing angle and maintain the critical state, thereby facilitating the printing of high‐quality UFNs. Controllable fiber diameter is crucial in regulating cell behavior. As shown in Figure 4c–e, under constant parameters (air pressure: 25 kPa, voltage: 3.5 kV, and height: 2 mm), collection speed increased from 500 to 900 mm min^−1^ and the swing angle of the jet increased, leading to a continuous decrease in fiber diameter. When the collection speed was 300 mm min^−1^, the jet had a slight whipping effect, leading to deposited crimped fiber. As shown in Figure s2a (Supporting Information) and Figure 4f, when other printing parameters remained constant, the jet swing angle increased with decreasing voltage, whereas the diameter of the deposited fibers was almost unchanged. The results demonstrated that with consistent extrusion flow rate and steady collection velocity, the change of the jet swing angle to a certain extent negligibly influenced fiber diameter. In conclusion, the voltage increase can reduce the swing angle of the jet but negligibly influences the consistency of fiber diameter.

Selected collection speeds with small jet swing angles were used to print orthogonal UFNs with different fiber spacings (150, 200, 300, and 500 µm) and 45° rhombus networks with fine fibers (15 µm) guiding cellular orientation and coarse fibers providing mechanical support (Figure 4g). MEW was used to print horizontal networks, which were then embedded into the optimal G5‐H0.5‐C0.5 hybrid biohydrogel to form horizontal biological reinforced concretes (HB‐150, HB‐200, HB‐300, and HB‐500). As shown in Figure 4h–j, compressive modulus decreased as fiber spacing increased, with HB‐150 having the highest compressive modulus at 484.39 kPa. Rhombus UFNs with different coarse fiber diameters (35 µm, 50 µm, 75 µm) were rolled into cylindrical scaffolds and embedded into the G5‐H0.5‐C0.5 hybrid biohydrogel to form vertical biological reinforced concretes (VB‐35, VB‐50, and VB‐75). As shown in Figure 4k,l, the compressive modulus of the vertical biological reinforced concretes increased as the support fiber diameter increased, with VB‐75 exhibiting the highest value at 527 kPa. Assembling VB‐75 and HB‐150 produced the BCCs with an overall compressive modulus of 542.56 kPa, close to the compressive modulus of natural cartilage.^[^ ^63^ ^]^

### 2.4 Effects of Biological Reinforced Concretes on Cell‐Orientated Growth and Cartilage ECM Deposition

In 3D culture, cells are provided with an environment similar to physiological conditions, allowing cells to grow in all directions, simulating the conditions of living tissues, and facilitating the observation of natural cellular behaviors.^[^ ^64^ ^]^ However, this relatively disorganized and chaotic growth pattern often proves insufficient for repairing tissues with precise structures, such as cartilage.^[^ ^65^ ^]^ The fiber alignment or organizational structure of the cartilage is essential for its proper function. During cartilage regeneration, the alignment of collagen is closely linked to cellular orientation. Cells exhibiting nondirectional growth or chaotic alignment, secrete a likely disorganized cartilage matrix. This correlation emphasizes the importance of cellular alignment in producing a structured and functional cartilage matrix. In the 3D microenvironment of biological reinforced concretes, cells interact with biohydrogel, undergoing proliferation, migration, aggregation, and division. It is essential to further explore the interactions between the UFNs and cells in this 3D environment.

**Figure 5:**
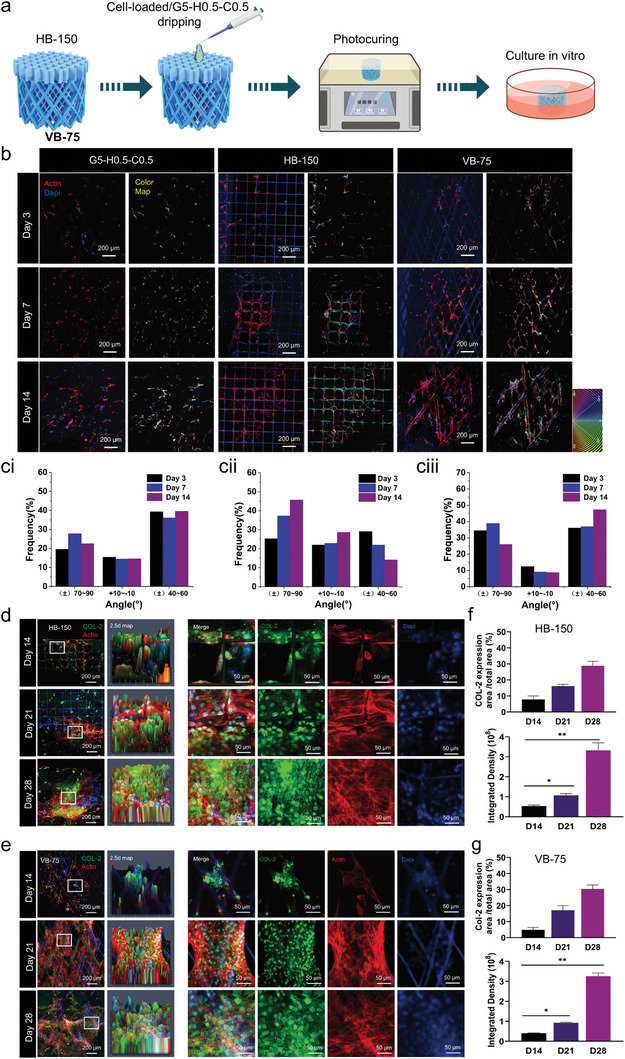
hBMSC migration, chondrogenesis, and matrix secretion in HB‐150 and VB‐75. a) Schematic depicting the experimental strategy for hBMSC migration and chondrocyte differentiation in HB‐150 and VB‐75 3D microenvironment. b) Confocal images of hBMSC migration performance in G5‐H0.5‐C0.5, HB‐150, and VB‐75 at 3, 7, and 14 days (F‐actin staining for cytoskeleton: red, DAPI staining for cell nucleus: blue). ci–ciii) The direction of cells in pure hybrid biohydrogel, HB‐150, and VB‐75 at 3, 7, and 14 days. d–e) Confocal images of COL‐2 expression in HB‐150 and VB‐75 at days 14, 21, and 28 (F‐actin: red, COL‐2: green; nucleus: blue). f–g) Quantitative evaluation of COL‐2 immunofluorescence integrated density and COL‐2 expression area/total area × 100 (%) in HB‐150 and VB‐75. Data are means ± SD. *p < 0.05, **p < 0.01, ***p < 0.001, and ****p < 0.0001.

In this section, validation studies were performed to ascertain the capabilities of HB‐150 and VB‐75 in guiding the directional growth of hBMSCs and facilitating the organized deposition of COL‐2. After suspending and mixing hBMSCs with the selected G5‐H0.5‐C0.5 biohydrogel, the mixture was dripped on orthogonal UFNs and multiscale 45° rhombus UFNs to form HB‐150 and VB‐75, respectively. Once full contact between the biohydrogel and UFNs was ensured, photocuring was performed, followed by in vitro culture (**Figure** **5**a). As shown in Figure 5b, confocal analysis after 3 days showed that a small number of cells began to migrate toward the UFNs, showing an inclination to align along them. By day 7, the directional migration of hBMSCs along the fibers became more pronounced, with cells tending to distribute along VB‐75 and HB‐150 and firmly adhering to the fibers. At day 14, within the field of view, the cells were almost entirely aligned along the fibers, showing directional growth. Additionally, the cells exhibited partial proliferation between the fibers, further indicating good biocompatibility of VB‐75 and HB‐150. By contrast, as a control group, in G5‐H0.5‐C0.5 hybrid biohydrogel, cells embedded within the biohydrogel exhibited “non‐directional growth” and “chaotic alignment”. This phenomenon is primarily due to the inherent properties of the biohydrogel matrix, which lacks the organized structure found in natural extracellular matrices. The biohydrogel does not possess specific topological cues or structured pathways to guide cell growth and tissue regeneration. It relies solely on limited biochemical cues and mechanical properties within the 3D microenvironment to direct physiological processes, such as cell proliferation, migration, aggregation, and differentiation. This can lead to more random or chaotic cell growth patterns. Indeed, this forms a stark contrast with a more structured 3D microenvironment.

To further demonstrate the role of UFNs as topological cues in inducing directional alignment of cells, a statistical analysis of cell polarity was conducted (Figure s3a‐c, Supporting Information). In the pure hybrid‐biohydrogel group, cells grew without a significant polarity trend. The quantitative statistics of cell growth polarity are shown in Figure 5ci. Over 14 days, the proportion of cells growing in the −10°≈10° and (±) 40°≈60° directions stabilized at ≈15% and 39%, respectively, while there was some fluctuation in the proportion growing in the (±) 70°≈90° direction, which is considered normal. Notably, in Figure S3d–i (Supporting Information), a clear trend of polarized cell growth can be observed with the addition of the UFNs. As shown in Figure 5cii, for the HB‐150 group, the proportion of cell growth polarity in the −10°≈10° and (±) 70°≈90° directions increased over time, from 21% and 25% on day 3 to 28% and 45% on day 14, respectively, while the growth polarity proportion in the (±)40°≈60° direction decreased correspondingly from 28% to 14%. In contrast, for the VB‐75 group, the growth polarity proportion in the (±) 40°≈60° direction increased over time from 35% on day 3 to 47% on day 14, with a noticeable decrease in the other two directions (Figure 5ciii). These results indicated that UFNs could guide cell migration and directional growth in a 3D microenvironment. The biological reinforced concretes (VB‐75 and HB‐150) not only mimic the 3D microenvironment of the ECM but also provide topological cues that imitate the specific structure of tissues, offering robust support for the directional growth of cells and biomimetic repair of tissues.

To delve deeper into the impact of biological reinforced concretes on the secretion of chondrogenic matrix by hBMSCs, a consistent method was employed to form VB‐75 and HB‐150. After culturing in a chondrogenic induction medium for 14, 21, and 28 days, followed by immunofluorescence staining, observations were made using a confocal microscope. By day 14, noticeable oriented deposition of COL‐2 was observed along the fibers on both HB‐150 and VB‐75 (Figure 5d,e). Over time, COL‐2 fluorescence intensity and area of COL‐2 expression distributed along the fibers on HB‐150 and VB‐75 showed a significant increase, markedly surpassing protein formation within the biohydrogel alone at similar time points (days 14 and 28) (Figure 5f,g). This suggests that compared to the chaotic generation of COL‐2 within the biohydrogel alone, topological cues within the biohydrogel's 3D environment not only guide the directional migration of hBMSCs but also enhance chondrogenic differentiation, inducing the orderly deposition of the cartilage matrix.

### 2.5 Evaluation of BCCs for Cartilage Defect In Vivo

**Figure 6:**
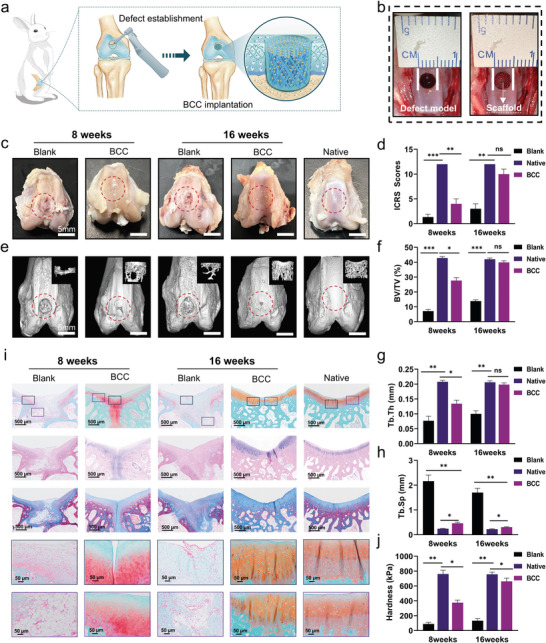
In vivo repair evaluations at 8‐ and 16‐weeks post‐surgery. a) Schematic diagram of animal experiments. b) Surgical procedure. c) Gross images of the repaired cartilage in blank, BCC, and native groups. d) Macroscopic scores according to the scoring system of the ICRS for gross observations. e) Micro‐CT 3D reconstruction images. f–h) Quantitative BV/TV ratio, Tb. Th, and Tb. Sp analysis of the new bone in the defects. i) Histological images of hematoxylin & eosin (H&E), safranin‐O/fast green, and Masson's trichrome stainings. j) Hardness in each group. Data are means ± SD. *p < 0.05, **p < 0.01, and ***p < 0.001.

To determine the effectiveness of biological reinforced concretes in accelerating the reconstruction of complex cartilage ECM structures, in vivo evaluations were conducted using a rabbit osteochondral defect model. The rabbits were randomly divided into three groups for evaluating osteochondral regeneration: blank, biomimetic cartilage construct (BCC), and native groups. Cylindrical defects (height: 4 mm, diameter: 4.5 mm) were created at the knee joint trochlear site (**Figure** **6**a,b). The repair of cartilage injury was assessed at 8 and 16 weeks post‐operation.

At predetermined time points, the experimental animals were euthanized, and specimens were collected for evaluation. The overall appearance of the defect areas in the specimens was observed and assessed (Figure 6c). Significant infection was not found in any group. At 8 weeks post‐operation, the defect shape was visible on the surface in the blank group, with the defect surface covered by a large amount of soft tissue, and no apparent cartilage formation was observed. At 16 weeks, the defect profile was visible in the injured area of the blank group, but the extent of the visible defect area decreased compared with that at 8 weeks. In the BCC group, by week 8, most of the defects were covered with newly formed cartilage tissue, although the surface was not smooth. By week 16, the defect in the BCC group had a gross appearance close to that of the native group and was almost filled with new cartilage, presenting a smooth surface that integrated with the surrounding tissue and making the boundary nearly indiscernible. The International Cartilage Repair Society (ICRS) score system was used to systematically evaluate the macroscopic results (Figure 6d).

Micro Computed Tomography (Micro‐CT) 3D reconstruction was then utilized to assess the regeneration of subchondral bone in the defect area (Figure 6e). At week 8, for the blank group, only a small amount of circumferential new bone formation was observed on the surface of the defect, with no new bone growth detected within the defect. By week 16, the effect of subchondral bone regeneration was slightly more pronounced. As shown in Figure 6f, the bone volume to total volume (BV/TV) ratio in the blank group increased from 7.10 ± 1.14% to 13.85 ± 0.86%. At 8 weeks, the BV/TV ratio in the BCC group was 27.67% ± 1.92%. In the native group, by comparison, the BV/TV ratio was 42.84% ± 0.933%. Remarkably, by week 16, the BV/TV ratio in the BCC group had increased to 39.68% ± 0.38%, closely approaching the native group's ratio of 41.99% ± 0.85%. These findings suggest a significant progression in the BCC group toward matching the structural characteristics of native tissue. By week 16, the defect area in the BCC group was densely filled with newly formed bone tissue, and the newly formed subchondral bone structure resembled that of native tissue (Figure 6e). The bone trabecular parameters shown in Figure 6g,h, including trabecular separation (Tb. Sp) and trabecular thickness (Tb. Th), further substantiate the advantages of the BCC group in repairing subchondral bone.

To assess the advantages of the BCC group in vivo regeneration, all samples were sectioned and performed histological evaluation (Figure 6i). The samples were subjected to hematoxylin & eosin, safranin O/fast green, and Masson's trichrome staining. Consistent with macroscopic observations, the blank group exhibited distinct structural disarray within the defect area, with no evident cartilage formation on the defect surface. Instead, the area was filled with fibrous connective tissue, with only a minimal amount of subchondral bone regeneration. In the blank group, the boundary between the injury site and surrounding tissue was delineated. By contrast, in the BCC group, the biomimetic design provided a better environment for cell proliferation, migration, and differentiation, promoting the development of tissue with normal structure and shape. At 16 weeks, cartilage repair was continuous and smooth, characterized by the regeneration of hyaline‐like cartilage tissue. In the superficial zone of the cartilage, chondrocytes were horizontally arranged, and concurrently, in the deep zone of the cartilage, radial chondrocyte columns similar to those in the native group were observed. This was significantly superior to the blank group. Furthermore, the biomechanical properties of cartilage treated with the blank, BCC, and native groups at 8 and 16 weeks were assessed using nanoindentation. At week 16, the newly formed cartilage in the BCC group exhibited a hardness level similar to that of the native group (Figure 6j), suggesting its potential for stability and optimal function within the joint environment.

In summary, the biological reinforced concrete demonstrated promising results in knee osteochondral regeneration, with the regenerated tissue exhibiting favorable structural and mechanical properties. This biomimetic design can serve as an effective repair strategy for addressing significant knee joint defects, facilitating the regeneration and reshaping of articular cartilage lesions.

### 2.6 Collagen Orientation and Distribution in the Reconstructed Cartilage

**Figure 7:**
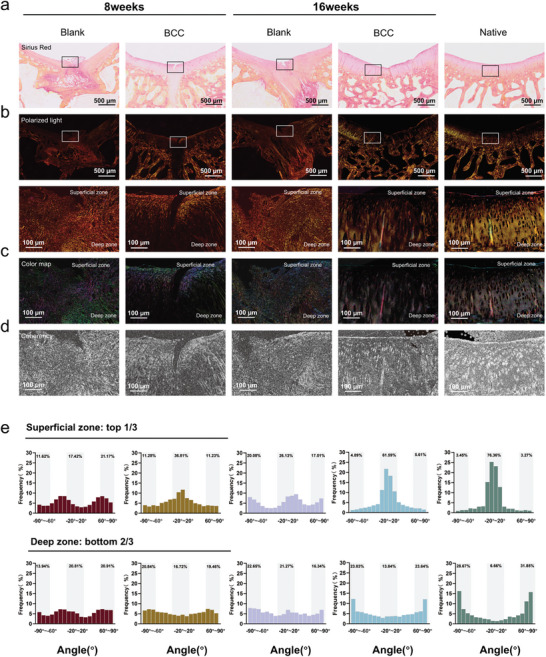
Quantitative analyses of collagen fiber distribution in each group at 8 and 16 weeks post‐surgery. a) Images of Sirius red staining. b) Polarized light images. c) Color map images of collagen fiber distribution. d) Coherency images of collagen fiber distribution. e) Comprehensive comparison of collagen fiber orientation within the superficial (top 1/3) and deep (bottom 2/3) zones.

In native cartilage, chondrocytes and collagen fibers in the superficial zone align parallel to the cartilage surface. As they transition into the deeper layers, their orientation shifts to a vertical alignment. This heterogeneous and distinctive collagen fiber orientation pattern plays a crucial role in the normal physiological function of joints, such as distributing stress uniformly and efficiently toward the subchondral bone.^[^ ^66^ ^]^ To evaluate the morphology and orientation of collagen in cartilage post‐repair, and further substantiate the potential of our biomimetic scaffold in cartilage regeneration, Sirius red staining was performed (**Figure** **7**a) on tissue sections and examined them under the polarized light microscope (Figure 7b).

The superficial (top 1/3) and deep (bottom 2/3) zones of regenerated tissue were chosen as distinct regions of interest for analysis. The results of collagen fiber orientation are depicted in Figure 7e. In the blank group, collagen fibers between the superficial and deep zones did not demonstrate physiologically oriented growth at 8 or 16 weeks. Instead, it revealed the formation of disorganized fibrous connective tissue filling the defect area exclusively. By contrast, at 8 weeks, the BCC group exhibited a trend in the superficial zone where collagen fibers tended to align between −20° and 20°, accounting for 36.81% of overall orientations. Additionally, 22.51% of collagen fibers aligned vertically in the range of (±) 60°–90°. Simultaneously, in the deep zone, 40.3% of collagen fibers were distributed in the range of (±) 60°–90°, while 16.72% of collagen fibers aligned between −20° and 20°.

At 16 weeks, this directional tendency became more pronounced. The proportion of parallel collagen fibers in the superficial zone reached up to 61.59%, and the alignment of collagen distributed in the range of (±) 60°–90° in the deep zone increased to 47.47%. These distribution rates approached those observed in native cartilage, which displayed proportions of 76.36% (−20° to 20°) in the superficial zone and 60.52% ([±] 60°–90°) in the deep zone. Notably, the results of color mapping (Figure 7c) and coherency analysis (Figure 7d) were consistent with these findings. The outcomes of this study suggest that the bionic cartilage scaffold can reconstruct the complex structure of cartilage.

## 3 Conclusion

In summary, the development of biological reinforced concrete has resulted in BCCs that effectively repair cartilage defects and reconstruct the complex ECM. A biocement recipe was systematically formulated that simulates the biochemical 3D microenvironment of cartilage ECM by screening a hybrid of three biohydrogels (GelMA, HAMA, and ChSMA). The biorebar regulates the mechanical properties of the BCCs to closely match those of natural cartilage, establishing anisotropic characteristics and stratified interfaces, as well as guiding directional cell migration and cartilage matrix deposition. The design approach of biological reinforced concrete allows for the customizable manufacture of biomimetic ECM constructs, facilitating clinical translation in tissue repair.

## 4 Experimental Section

### Preparation of Hybrid Biohydrogels

Three kinds of freeze‐dried biohydrogels (EFL‐GM‐30, Mw: 100–200 kDa; EFL‐ChSMA‐001, Mw: 65–85 kDa; and EFL‐HAMA‐150k, Mw: 100–200 kDa) were obtained from Suzhou Intelligent Manufacturing Research Institute (Suzhou, China). To prepare the pre‐polymerization solution of the hybrid biohydrogels, the three biohydrogels were weighed separately according to concentration ratios and dissolved in a phosphate‐buffered saline (PBS) solution containing 0.3% lithium phenyl‐2,4,6‐trimethyl benzoate (Suzhou Intelligent Manufacturing Research Institute, 0.5% (w/v), purity: 99.8%). The prepolymer solution was filtered and sterilized using a 0.22 µm filter, and cured by irradiation with blue light (405 nm; intensity: 30 mW cm^−2^) for 30 s. Therefore, seven formulations were developed (Table 1).

### Design and Fabrication of UFNs

Before printing the UFNs, network structures with different porosities were designed by adjusting the printing path. These included: (1) orthogonal fine fiber networks with fiber spacings of 150, 200, 350, and 500 µm; (2) multiscale rhombus networks with a cross‐angle of 45°, ultrafine fiber spacing of 150 µm, and thick fiber spacing of 500 µm. For both structures, 10 layers of ultrafine fibers were printed, while the rhombus‐network thick fibers consisted of 2 layers. A high‐resolution MEW printer (Engineering for Life Co. Ltd., EFL, BP6602) was used to fabricate the UFNs. PCL pellets (CAPA6800, Perstorp Ltd., Sweden, Mw: 8000 g mol^−1^, melting point: 60 °C) were added into a syringe. The nozzle (size: 150 µm) and a material cylinder were preheated to 120 °C. Printing parameters are shown in **Table** **2**.

**Table 2:** Printing parameters.

Fiber diameter [µm]	Printing distance [mm]	Speed [mm min^−1^]	Air pressure [kPa]	High voltage [kV]
15	2	900	75	3.07
35	2	900	350	3.26
50	2	400	350	2.60
75	2	180	350	1.89

### Scanning Electron Microscopy of the UFNs and Biohydrogel Porosity

The microstructures of UFNs and freeze‐dried solidified biohydrogels, which were cylindrical with 5 mm height and 5 mm diameter, were sputter‐coated with gold for 2 min under vacuum conditions using a sputter coater (Ion Sputter E‐1045, Hitachi, Tokyo, Japan). The UFNs were then scanned by SEM at an acceleration voltage of 3 kV (SEM, Hitachi SU‐8010, Japan). ImageJ software was used to estimate the fiber diameter of the UFNs and the pore sizes of the biohydrogels (*n* = 30–40).

### Preparation of Biological Reinforced Concretes for BCCs

The UFNs were immersed in a 3 mol L^−1^ sodium hydroxide solution for ≈2 h to enhance the hydrophilicity. Following etching, the UFNs were sequentially washed with deionized water 3 to 5 times to achieve a pH of ≈7.4. After the hydrophilic treatment, the UFNs were cut into cylinders (diameter: 4 mm) using a biopsy knife. The first layer of the BCCs contained 16 layers of orthogonal networks (thickness: 0.8 ± 0.2 µm). The second layer was prepared by rolling the multiscale rhombus networks into a cylinder (diameter: 4 mm) and then cut into a cylinder (length: 3.2 mm). The two layers were placed in a polydimethylsiloxane custom mold individually to produce a cylinder (height: 4 mm, diameter: 4 mm). Then the hybrid biohydrogel prepolymer was poured to completely infiltrate the construct. Finally, the composite constructs were cured for 30 s by blue light irradiation (wavelength: 405 nm, intensity: 30 mW cm^−2^).

### Mechanical Test

The hybrid biohydrogels, prepared as cylinders with a height of 5 mm and a diameter of 8 mm, were used as test samples. These samples were tested for compressive modulus at a speed of 2 mm min^−1^ using a universal tensile machine (UTM2203, China) equipped with a 20 N load cell. To simulate the in vivo situation, the BCCs, as well as the first‐layer and second‐layer biological reinforced concretes, were prepared and immersed in PBS at 37 °C for 1–2 d. Compressive modulus was tested with a universal tensile machine at a speed of 1 mm min^−1^. Compressive modulus was calculated using a stress‐strain curve at 12%–15% strain(n ≥3). The photorheological behavior of the biohydrogels was tested with a rheological instrument (MCR102, Anton Paar, Austria). The biohydrogel samples were cured by blue light irradiation after 24 s.

### Cell Harvest and Culture

The hBMSCs were obtained from Zhejiang Meisen Technology Co., Ltd. These cells were cultured in high‐glucose Dulbecco's modified Eagle's medium (DMEM, Sigma‐Aldrich, USA) supplemented with 10% fetal bovine serum (Hyclone, USA) and 1% penicillin‐streptomycin (Sigma‐Aldrich, USA). The incubation was conducted in a humidified atmosphere at 37 °C with 5% CO_2_. Primary hBMSCs from passages 3–5 were used for the subsequent experiments.

### Hybrid Biohydrogel‐Based 3D Culture

For hybrid biohydrogel‐based 3D‐cultured studies, hBMSCs were collected and resuspended in a 37 °C gel solution (1 × 10^5^ cells per 100 µL). Uniformly dispense the cell‐loaded hybrid biohydrogel onto the sterile curing ring (EFL, Suzhou, China) using a pipette. After photocuring with blue light was complete, the curing ring was transferred with the cured 3D samples to a 24‐well plate for culture. Then, 2 mL medium was added to each well to merge the curing ring, and the medium was changed every 3 days.

### Cell Viability in Hybrid Biohydrogel 3D Culture

The proliferation of hBMSCs in 3D culture was evaluated by the cell counting kit‐8 assay (CCK‐8, Dojindo, Japan). Briefly, after 1, 7, and 14 days of in vitro culture, the liquid in each well was discarded, CCK‐8 solution was added under manufacturer's guidelines, and incubated at 37 °C in 5% CO_2_ for 4 h. Then, collected and transferred solutions to a new 96‐well plate and measured optical density (OD) at 450 nm using a microplate reader (Multiskan MK3, Thermo, USA).

For the live/dead assay, after 1, 7, and 14 days in vitro 3D culture, samples were washed with PBS three times, then stained with Calcein‐AM/PI (Solarbio, Beijing, China) in the dark at 37 °C following the instructions. The stained samples were observed immediately by confocal laser scanning microscopy (CLSM, Zeiss LSM 900, Carl Zeiss, Germany).

### Cell Integrin‐β1, Focal Adhesion Kinase, Neural Cadherin, Neural Cell Adhesion Molecule 1 Expression in Hybrid Biohydrogel 3D Culture

After 7 days of 3D culture in vitro, the cell‐biohydrogel complexes were fixed with 4% paraformaldehyde for 30 min; permeabilized with 0.1% TritonX‐100 for 15 min; blocked with 5% (w/v) bovine serum albumin (Sigma‐Aldrich, USA) for 2 h at 24 °C; and incubated at 4 °C overnight with primary antibodies against integrin‐β1 (1:200, ab30394, Abcam), FAK 1:400, 12636‐1‐AP, Proteintech), N‐cadherin (1:100, 66219‐1‐Ig, Proteintech), and NCAM1 (1:200, 14255‐1‐AP, Proteintech. Then the samples were incubated with 1:500 dilution of secondary antibodies at room temperature for 1 h: Alexa Fluor 488 (ab150113, Abcam), Alexa Fluor 488 (ab150077, Abcam), and Alexa Fluor 647(ab150075, Abcam). Each group was costained with 70 nmol rhodamine‐phalloidin (Cytoskeleton, USA) for 2 h and 4′,6‐diamidino‐2‐phenylindole (DAPI) for 10 min to stain nuclei. At each interval, samples were washed with PBS three times. All experimental groups were imaged immediately by CLSM.

To study the expression of genes involved in cell migration, aggregation, and condensation in 3D culture at day 7, relative gene expression of integrin‐β1, FAK, N‐cadherin, and NCAM1 were analyzed by reverse transcriptase real‐time PCR (qRT‐PCR). All groups were incubated in a medium containing collagenase II (EFL‐ColII‐DE‐001) and hyaluronidase (EFL‐HA‐DE‐001) to facilitate biohydrogel degradation. The samples were then centrifuged, after which the supernatants were discarded. Total RNA was extracted with TRIzol reagent (Invitrogen, USA) and RNA from each sample was reverse transcribed to cDNA using Hifair II Strand cDNA Synthesis Kit (Yeasen, China). qRT‐PCR was performed using SYBR Green Master Mix kit (Yeasen, China) with an Applied Biosystems 7500 Fast Real‐Time PCR System (Thermo Fisher Scientific). Glyceraldehyde‐3‐phosphate dehydrogenase (GAPDH) was used for normalizing gene expression. Primer sequences are listed in Table S1 (Supporting Information). The change in relative expression in the target gene was determined by normalizing to GAPDH expression using the 2^−△△Ct^ method.

### Cell Chondrogenic Differentiation Ability in Hybrid Biohydrogel 3D Culture

Chondrogenic induction medium contained DMEM with 1% penicillin‐streptomycin, 100 µg mL^−1^ sodium pyruvate (Solarbio, Beijing, China), 40 µg mL^−1^ L‐proline (Solarbio, Beijing, China), 50 µg mL^−1^ L‐ascorbic acid‐2‐phosphate (Sigma‐Aldrich, USA), 100 nM dexamethasone (MedChemExpress, USA), 1% insulin–transferrin–selenium (ITS) (Cyagen, Suzhou, China), and 10 ng mL^−1^ TGF‐β3 (Novoprotein, Suzhou, China). For all groups, the medium was changed every 3 days.

After 3D culture in different groups of hybrid biohydrogels for 28 days was complete, the samples were fixed with 4% paraformaldehyde (Beyotime, China), stained with Toluidine Blue (Solarbio, Beijing, China) and Alcian Blue Stain kits (Beyotime, Beijing, China) separately.

After 28 days of chondrogenic induction, samples were washed with PBS, fixed with 4% paraformaldehyde (Servicebio, China) for 30 min, permeabilized with 0.1% Triton X‐100 for 15 min, blocked with 10% goat serum at room temperature for 1 h, incubated overnight at 4 °C with primary antibodies against collagen‐2 (1:200, ab34712, Abcam), and with incubated Alexa Fluor 488 secondary antibody (1:500, ab150077, Abcam) at room temperature for 1 h. All groups were stained with DAPI for 10 min. Images were captured using CLSM.

The expression of genes involved in chondrogenesis: COL‐2, ACAN, and SOX‐9, was measured by qRT‐PCR on days 14 and 28. The primer sequences are listed in Table S1 (Supporting Information).

### Morphology and Migration Characteristics in Biological Reinforced Concretes

To evaluate the effect of UFNs on cell morphology and migration in 3D culture, UFNs were immersed in 75% ethanol for 2 h and illuminated on both sides with ultraviolet light, washed three times with PBS, and incubated in DMEM for 1 h. The hBMSCs were collected and resuspended in a 37 °C‐biohydrogel solution ((1 × 10^6^ cells per 100 µL). Uniformly dispense the cell‐loaded selected biohydrogel onto the UFNs. After blue light photocuring was complete, the composite was transferred to a 24‐well plate (Corning 3337, USA) with DMEM for culture. The medium was changed every 3 days. After 3, 7, and 14 days of coculture, the samples were stained with rhodamine‐phalloidin to evaluate the morphology of the cytoskeleton using CLSM. The orientation analysis of cell distribution was performed using ImageJ.

### Collagen Type II Expression in Biological Reinforced Concretes

The UFNs were sterilized and pretreated according to the methods described. After hBMSC‐loaded biohydrogel was seeded and photocured on UFNs, the composite was cultured in a 24‐well plate with a chondrogenic induction medium. The medium was changed every 2 days. After chondrogenic induction for 14, 21, and 28 days, the samples were collected, stained with the chondrocyte marker collagen‐2 (1:200, ab34712, Abcam), and observed using CLSM.

### Surgical Procedure

This study was conducted in accordance with ethical principles, and approved by the Ethical Committee Board of The Second Affiliated Hospital of Zhejiang University School of Medicine.Male New Zealand white rabbits (weight: 3.0–3.5 kg), provided by Zhejiang Academy of Medical Sciences, were used to construct osteochondral defect models. The rabbits were housed in a comfortable environment and supplied with abundant water and food. After the rabbits were anesthetized, a midline incision was performed. The attachment of the medial collateral ligament was cut off to dislocate the patella and expose the knee joint. A cylindrical defect (height: 4 mm, diameter: 4 mm) was created with a Micro Power System (Stryker, USA) in the center of the trochlear groove. Then, a BCC was implanted into the defect. After the operation, all rabbits had regular rest, unrestricted movement, and plenty of food and water. The rabbits were euthanized on weeks 8 and 16 post‐operation. Samples were collected, and macroscopic observations were scored according to the International Cartilage Repair Society (ICRS) (Table S2, Supporting Information).

### Micro‐CT Evaluation

All rabbit knee samples were fixed with 4% polyformaldehyde for 48 h. Micro‐CT (Milabs, Netherlands) scanning was applied to evaluate the reconstruction of the osteochondral defect. A cylindrical region (height: 4 mm, diameter: 4 mm) was defined to analyze regeneration within the defect site. The bone tissue volume/total tissue volume (BV/TV), trabecular thickness (Tb. Th), and trabecular separation (Tb. Sp) were quantified.

### Histological and Hardness Evaluation

The harvested samples were fixed with 4% paraformaldehyde, decalcified with 10% ethylenediaminetetraacetic acid for 8 weeks on a shaking table at 37 °C, routinely dehydrated, embedded in paraffin blocks, and sectioned into 5‐µm slices. Safranin‐O/Fast Green, hematoxylin and eosin (H&E), Masson's trichrome, and picrosirius red staining were performed according to standard protocols. Stained slides were observed and scanned using a Slideview system (VS200, Olympus, Japan). Picrosirius red sections were observed under a polarized light microscope. The hardness of the central part of the blank, BCC, and native groups was tested at 8 and 16 weeks post‐surgery using Hysitron TI Premier (BRUKER, Germany). During the test, the samples were maintained in a hydrated state with PBS.

### Evaluation of Collagen Orientation and Distribution

Picrosirius red‐stained samples were imaged using VS200 polarized light mode. Analysis of collagen orientation and coherency and reconstruction of the color map were performed using ImageJ software.

### Statistical Analysis

Quantitative data are presented as mean ± standard deviation (SD). Data were analyzed by GraphPad Prism 9 and Origin 2021 using one‐way analysis of variance (ANOVA) with Tukey's *post hoc* test. *p* < 0.05 was considered statistically significant.

## Conflict of Interest

The authors declare no conflict of interest.

## Supporting information



Supporting Information

## Data Availability

Research data are not shared.
