# Supplementary material for: Biological Reinforced Concrete for Cartilage Repair With 3D Printing
Source: Adv Sci (Weinh). 2025 Feb 25;12(16):2416734. doi: 10.1002/advs.202416734 (PMC12021066; doi:10.1002/advs.202416734)
Supplement: Supplementary file 1 — Supporting Information [file ADVS-12-2416734-s001.docx]

## Supplementary Materials for

## Biological reinforced concrete for cartilage repair with 3D printing

YueweiChen^1,2,†^, Tao Fu^3, 4, †^,Zhongfei Zou^7, †^,Yanming Liu^3^, Jianguo Zhu^6^,

BinhongTeng^5^, KeYao^2^, Haibin Li^2^, JiachunLi^1,^*, Zhijian Xie^4,^*, Yong He^2,^*

^1^School of Mechanical Engineering, Guizhou University, Guiyang, 550025, China.

^2^State Key Laboratory of Fluid Power and Mechatronic Systems & LiangzhuLaboratory, School of Mechanical Engineering, Zhejiang University, Hangzhou310027, China.

^3^Department of Oral and Maxillofacial Surgery, The Second Affiliated Hospital of Zhejiang University School of Medicine, School of Stomatology and Key Laboratory of Oral Biomedical Research of Zhejiang Province, Hangzhou, Zhejiang, 310000, China.

^4^Stomatology Hospital, School ofStomatology, Zhejiang University School of Medicine, Zhejiang ProvincialClinical Research Center for Oral Diseases, Key Laboratory of Oral BiomedicalResearch of Zhejiang Province, Cancer Center of Zhejiang University, EngineeringResearch Center of Oral Biomaterials and Devices of Zhejiang Province, Hangzhou, 310000, China.

^5^Department of Orthodontics, TheSecond Affiliated Hospital of Zhejiang University School of Medicine, Hangzhou,Zhejiang, 310000,China.

^6^Department of Urology, GuizhouProvincial People’s Hospital, The Affiliated Hospital of Guizhou  University, Guiyang, Guizhou Province,550002, China.

^7^Schoolof Mechanical Engineering, Guizhou Institute of Technology, Guiyang,550003.China.

*Corresponding authors.

†Coauthor

E-mail:yongqin@zju.edu.cn (Yong He)

E-mail:[xzj66@zju.edu.cn](mailto:xzj66@zju.edu.cn)(Zhijian Xie)

E-mail:jcli@gzu.edu.cn (Jiachun Li)

**The PDF file includes:**

**Table S1.** Primers for RT-PCR analysis

**Table S2.** ICRS macroscopic morphology scoring system

**Figure s1.** Photorheology of hybrid biohydrogels

**Figure s2.** Effect of voltage on jet swing Angle

**Figure s3.** The proportion of polar growth of 3D cultured BCCs in three directions: -10°~10°, (±) 40°~60°, (±) 70°~90°

**Table S1.** Primers for RT-PCR analysis

| **Gene** | **Forward primer (5'-3')** | **Reverse primer (5'-3')** |
| --- | --- | --- |
| GAPDH  Integrin β1  FAK  NCAM1  N-Cadherin  COL2A1  ACAN | GATTCCACCCATGGCAAATTC  CCTACTTCTGCACGATGTGATG  GCGTCTAATCCGACAGCAACA  GCTGGACAAAGGATGGGGAA  GATCAGCGTGCTGGACGTAGAT  TGGACGATCAGGCGAAACC  TGCATTCCACGAAGCTAACCTT | CTGGAAGATGGTGATGGGATT  CCTTTGCTACGGTTGGTTACATT  ACTGCCTCGAGAGAGTCTCACAT  TTTGAGGTGGATGGTCGCAT  TGGTCCCTACCGGAGTGTCTTC  GCTGCGGATGCTCTCAATCT  GACGCCTCGCCTTCTTGAA |
| SOX-9 | AGCGAACGCACATCAAGAC | CTGTAGGCGATCTGTTGGGG |

**Table S2.** ICRS macroscopic morphology scoring system

| Cartilage Repair Assessment ICRS | Points |
| --- | --- |
| Degree of defect repair |  |
| In level with surrounding cartilage | 4 |
| 75% repair of defect depth | 3 |
| 50% repair of defect depth | 2 |
| 25% repair of defect depth | 1 |
| 0% repair of defect depth | 0 |
| Integration to border zone |  |
| Complete integration with surrounding cartilage | 4 |
| Demarcating border < 1 mm | 3 |
| Three-fourth of graft integrated, one-fourth with a notable border > 1 mm width | 2 |
| Half of graft integrated with surrounding cartilage, and half with a notable border > 1 mm | 1 |
| Form no contact to one-fourth of graft integrated with surrounding cartilage | 0 |
| Macroscopic appearance |  |
| Intact smooth surface | 4 |
| Fibrillated surface | 3 |
| Small, scattered fissures, or cracks | 2 |
| Several, small, or few but large fissures | 1 |
| Total degeneration of grafted area | 0 |
| Overall repair assessment |  |
| Grade I: normal | 12 |
| Grade II: nearly normal | 11-8 |
| Grade III: abnormal | 7-4 |
| Grade IV: severely abnormal | 3-1 |


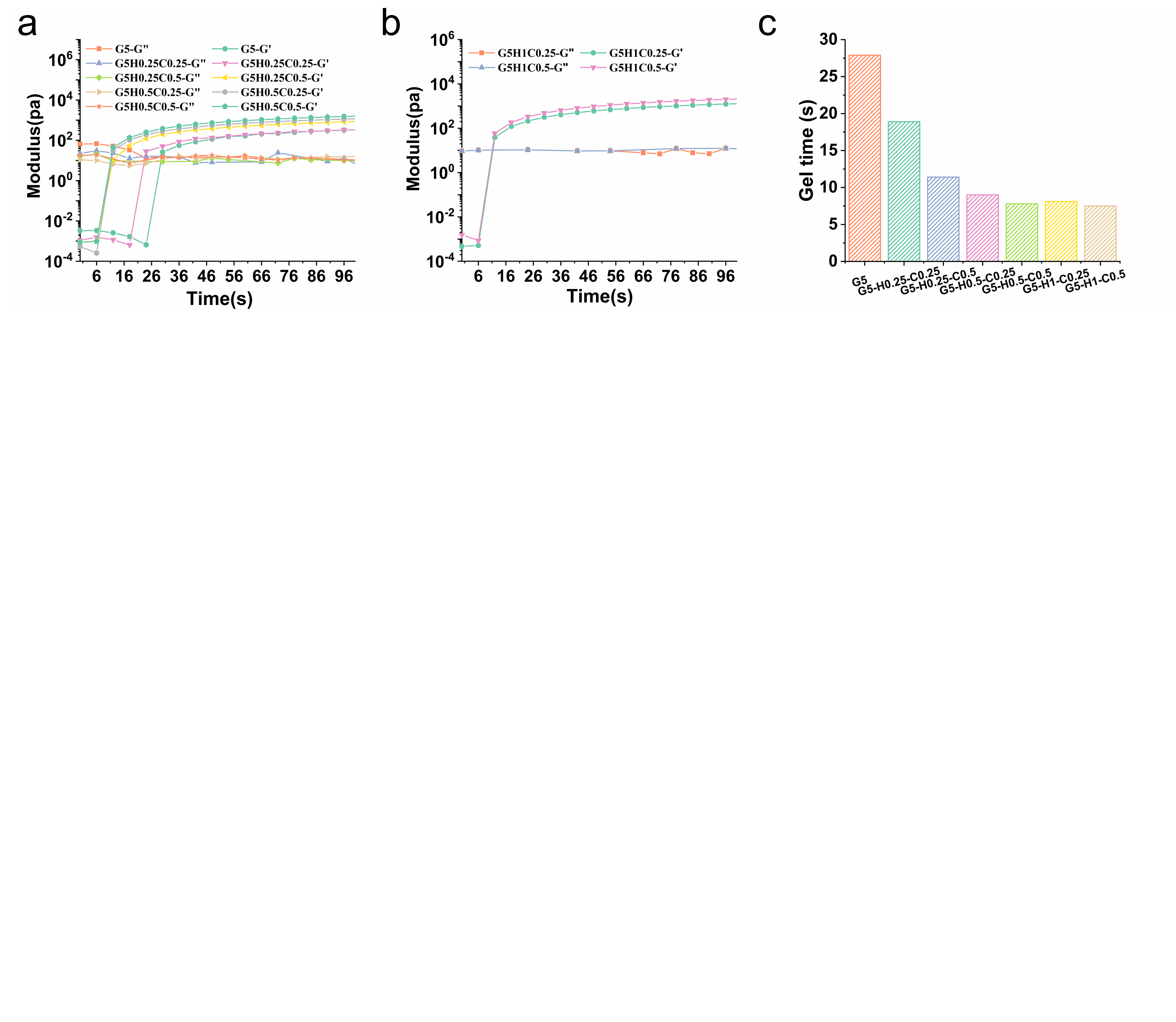


**Figure s1.** **Photorheology of hybrid biohydrogels:** **a)** Group 1-5 photorheology curves. **b)** Group 6-7 photorheological curves. **c)** Gel times for all groups.


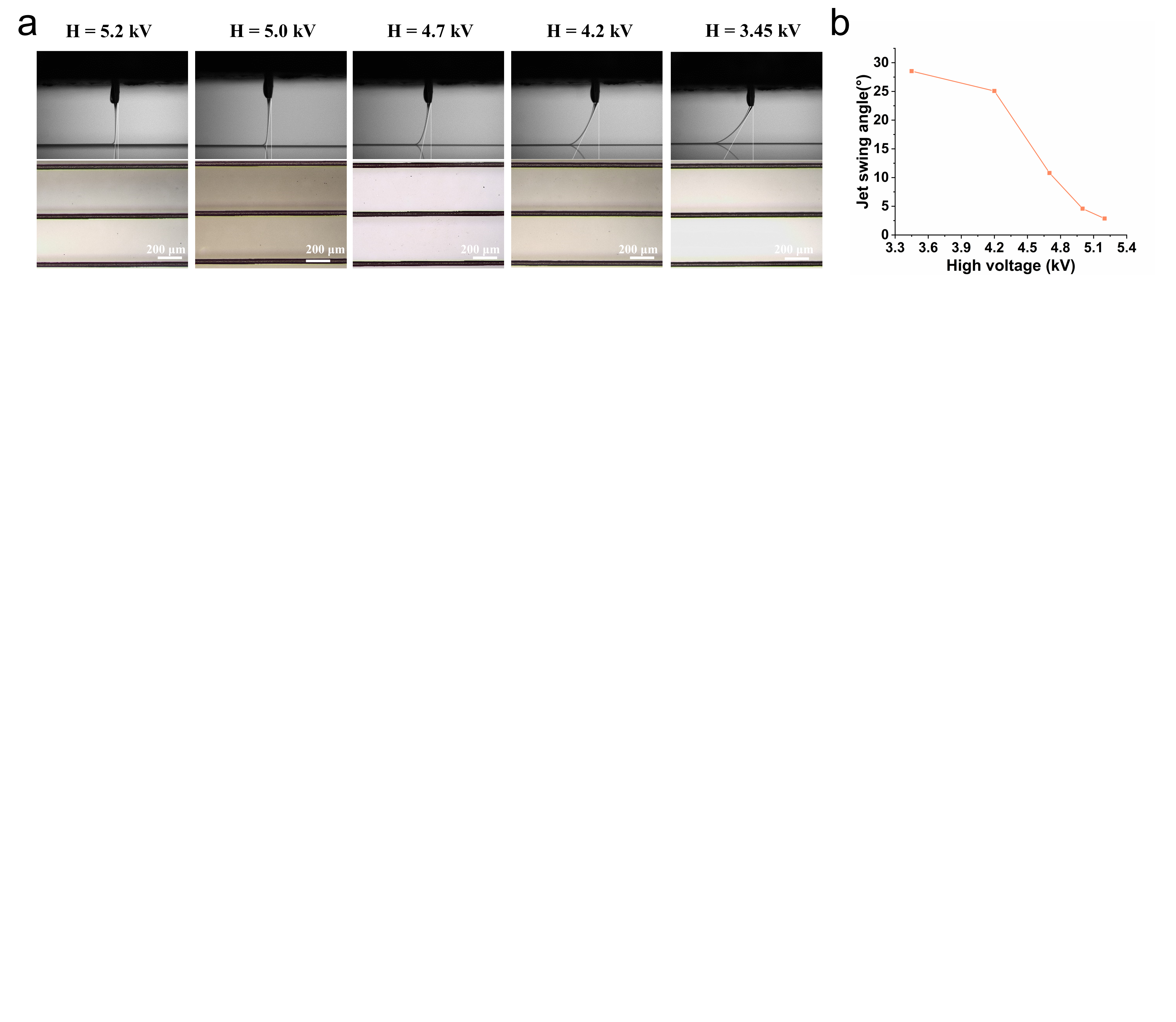


**Figure s2. Effect of voltage on jet swing angle.** **a)** Image of jet swing angle and deposited fiber state at different voltages. **b)** Jet swing angle at different voltages.


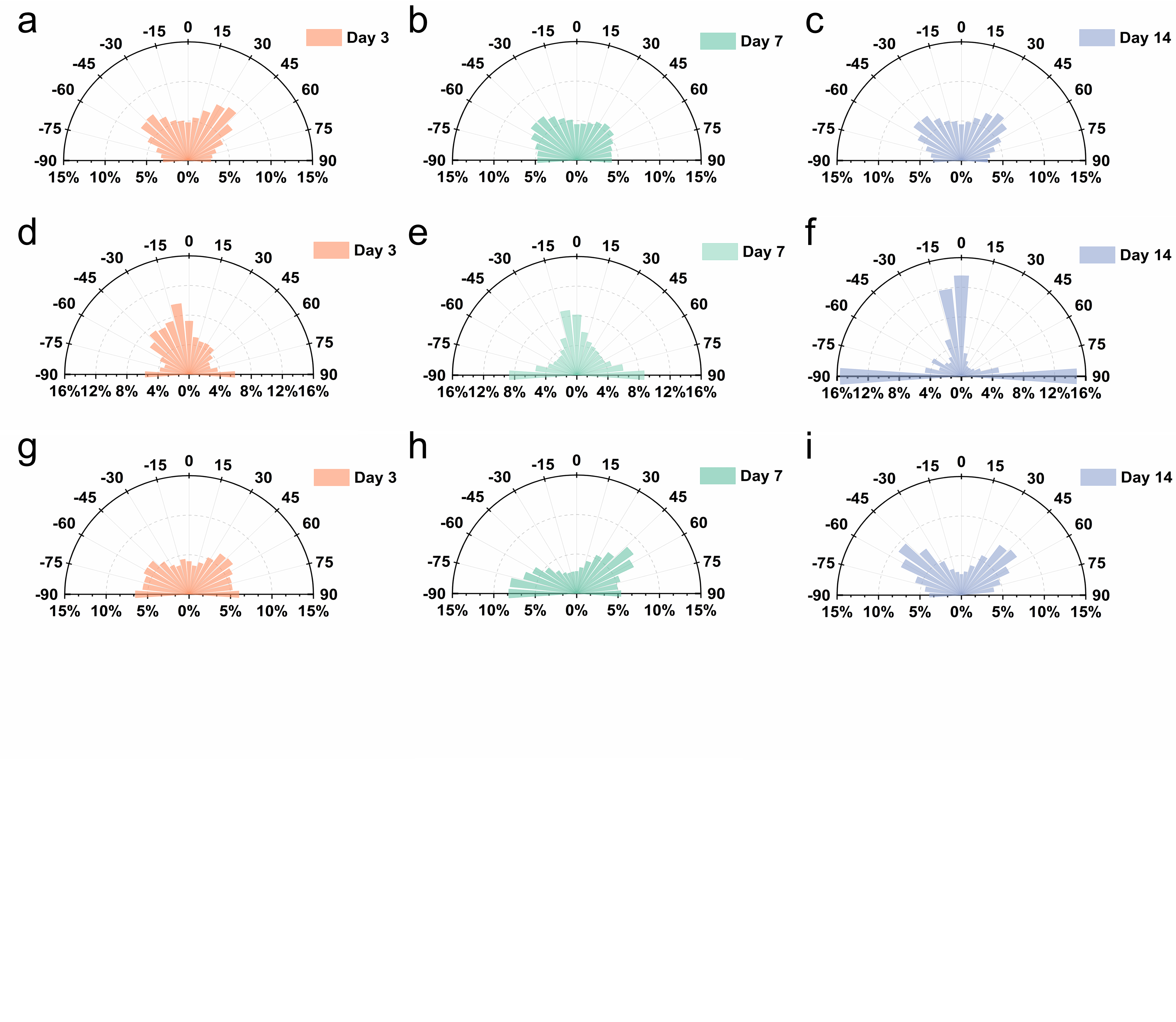


**Figure s3.** **The proportion of polar growth of 3D cultured BCCs in three directions: -10°~10°, (±) 40°~60°, (±) 70°~90°**. **a-c)** G5-H0.5-C0.5 hydrogel; **d-f)** HB-150 bioconcretes; **g-i)** VB-100 bioconcretes.
